# Supplementary material for: Some Like it Hot: Efficiency of the Type III Secretion System has Multiple Thermosensitive Behaviours in the Pseudomonas syringae Complex
Source: Mol Plant Pathol. 2025 Dec 10;26(12):e70170. doi: 10.1111/mpp.70170 (PMC12696027; doi:10.1111/mpp.70170)
Supplement: Supplementary file 1 — Figure S1: Variance of the mean area under conductivity progress curve (AUCPC) values obtained for each 39 modalities avrB‐expressing strain × temperature. Conductivity was measured over time in Arabidopsis thaliana Col‐0 leaf disks infiltrated with the different strains under different temperature conditions. Number of replicated experiments for each strain varied from 3 up to 27. Linear regression trend curve was fitted to the data, with y = 323.89x − 150932 and R 2 = 0.8588, showing a positive correlation between mean AUCPC and their variance (Spearman's coefficient = 0.91; p < 0.05). [file MPP-26-e70170-s002.pdf]

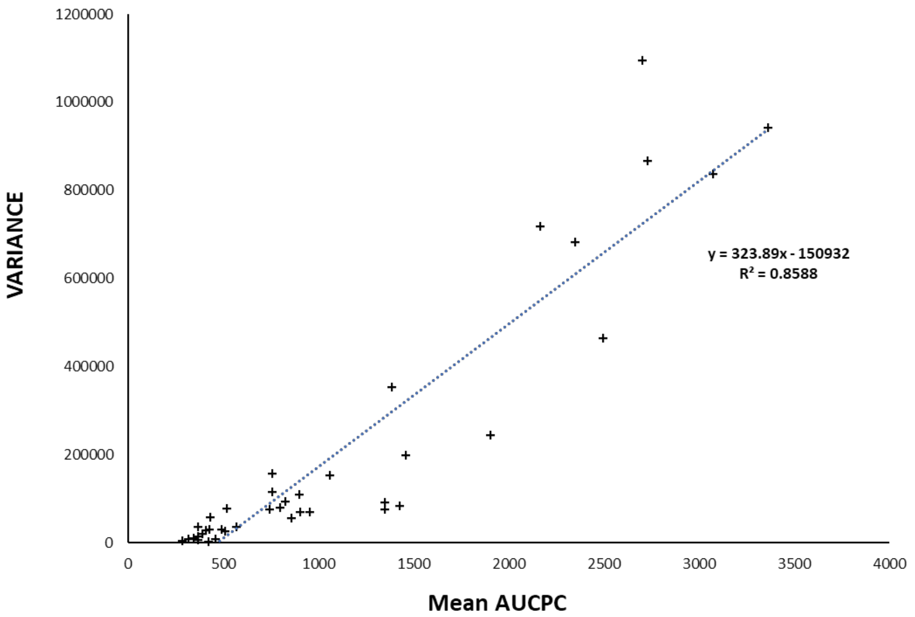

Figure S1. Variance of the mean area under conductivity progress curve (AUCPC) values obtained for each 39 modalities *avrB*-expressing strain x temperature. Conductivity was measured over time in *Arabidopsis thaliana* Col-0 leaf disks infiltrated with the different strains under different temperature conditions. Number of replicated experiments for each strain varied from 3 up to 27. Linear regression trend curve was fitted to the data, with y=323.89x – 150932 and R^2=0.8588, showing a positive correlation between mean AUCPC and their variance (Spearman's coefficient = 0.91; $p < 0.05$).
