# Supplementary material for: Some Like it Hot: Efficiency of the Type III Secretion System has Multiple Thermosensitive Behaviours in the Pseudomonas syringae Complex
Source: Mol Plant Pathol. 2025 Dec 10;26(12):e70170. doi: 10.1111/mpp.70170 (PMC12696027; doi:10.1111/mpp.70170)
Supplement: Supplementary file 2 — Figure S2: avrB expression among the mutant strains. RNA was extracted from bacterial suspensions grown for 5 h at 24°C in liquid hrp‐inducing medium (HIM). (a) Real‐time PCR was performed using rpoD as the housekeeping gene. Expression levels were established using the mean normalised expression (MNE). Error bars represent standard error. Data represent the mean of three independent biological replicates. (b) Scatterplot of hpi 50% versus avrB expression (MNE) showing the absence of correlation between the two variables according to the Pearson correlation. [file MPP-26-e70170-s001.pdf]

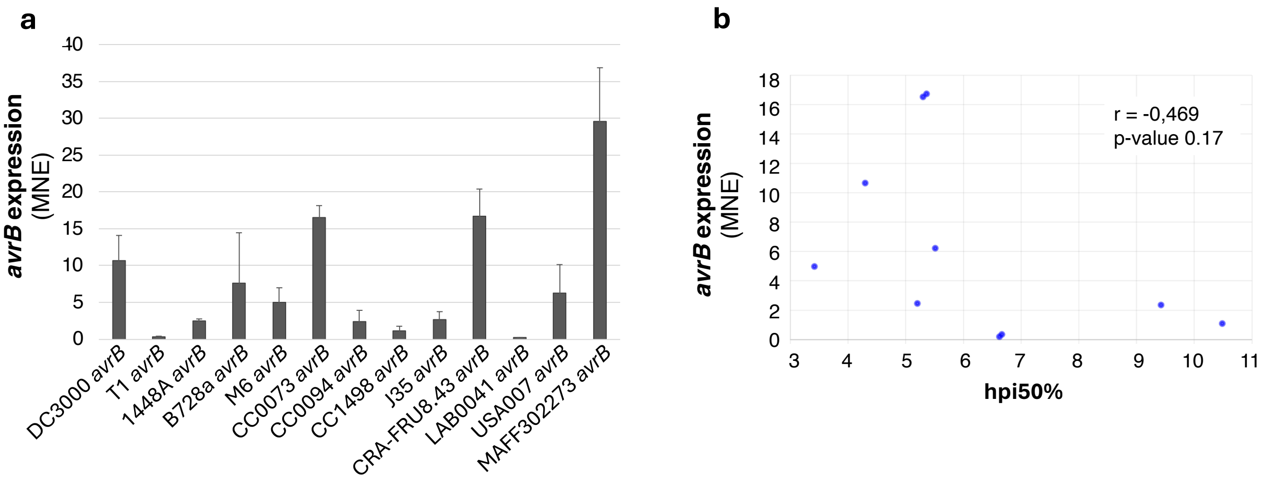


**Figure S2. *avrB* expression among the mutant strains.** RNA was extracted from bacterial suspensions grown for 5 hours at 24°C in liquid *hrp*-inducing medium (HIM). (**a**) Real-time PCR was performed using *rpoD* as the housekeeping gene. Expression levels were established using the Mean Normalized Expression (MNE). Error bars represent standard error. Data represent the mean of three independent biological replicates. (b) Scatterplot of hpi50% versus *avrB* expression (MNE) showing the absence of correlation between the two variables according to the Pearson correlation.
