# Supplementary material for: Some Like it Hot: Efficiency of the Type III Secretion System has Multiple Thermosensitive Behaviours in the Pseudomonas syringae Complex
Source: Mol Plant Pathol. 2025 Dec 10;26(12):e70170. doi: 10.1111/mpp.70170 (PMC12696027; doi:10.1111/mpp.70170)
Supplement: Supplementary file 3 — Figure S3: In vitro growth curves for DC3000 avrB (a), USA007 avrB (b) and CC0094 avrB (c). Bacterial growth was measured for 24 h at 18°C (blue), 24°C (pink) and 28°C (red). Overnight grown bacteria were resuspended in liquid KB medium supplemented with kanamycin (50 μg/mL) and rifampicin (50 μg/mL, for DC3000 avrB only) at an initial load of 107 CFU/mL (optical density measured at 600 nm = 0.01). Data represent one single representative biological replicate with all strains and temperature conditions evaluated simultaneously with six technical replicates each. Error bars represent standard error. [file MPP-26-e70170-s006.pdf]

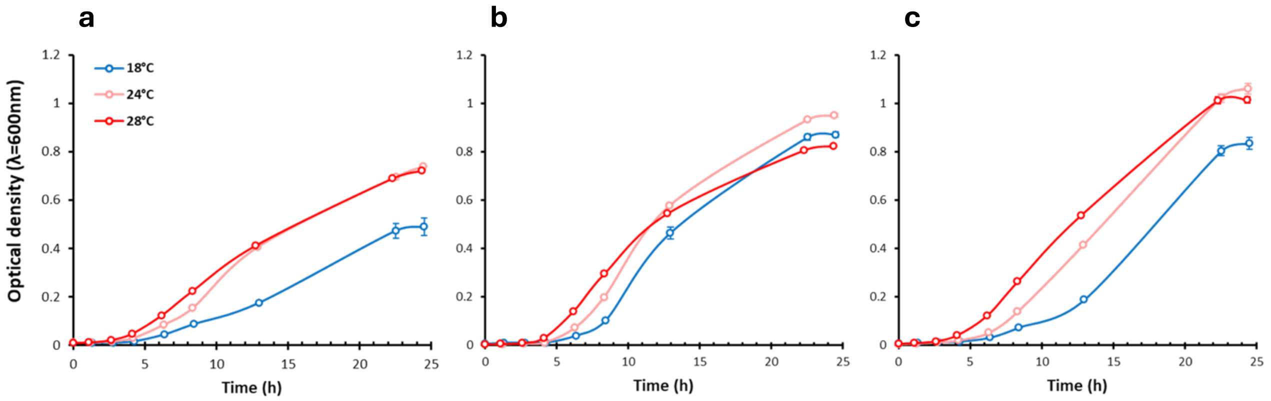

Figure S3. ***In-vitro* growth curves for DC3000 *avrB* (a), USA007 *avrB* (b) and CC0094 *avrB* (c).** Bacterial growth was measured for 24 hours at 18°C (blue), 24°C (pink) and 28°C (red). Overnight grown bacteria were resuspended in liquid KB medium supplemented with kanamycin (50µg/mL) and rifampicin (50µg/mL, for DC3000 *avrB* only) at an initial load of 10^7 CFU/mL (optical density measured at 600nm = 0.01). Data represent one single representative biological replicate with all strains and temperature conditions evaluated simultaneously with six technical replicates each. Error bars represent standard error.
