# Supplementary material for: Some Like it Hot: Efficiency of the Type III Secretion System has Multiple Thermosensitive Behaviours in the Pseudomonas syringae Complex
Source: Mol Plant Pathol. 2025 Dec 10;26(12):e70170. doi: 10.1111/mpp.70170 (PMC12696027; doi:10.1111/mpp.70170)
Supplement: Supplementary file 4 — Figure S4: Electrolyte leakage curves over time obtained post‐infiltration for CC0094 in A. thaliana Col 0 (a) and CRA‐FRU 8.43 in Actinidia arguta (b). Infiltrated leaf disks were incubated at 18°C (blue) and 28°C (red). Conductivity following infiltration of the mock treatment (10 mM MgCl2) and incubation at 18°C is represented by the black dotted line. Data represent one single representative biological replicate with all strains and temperature conditions evaluated simultaneously with three technical replicates each. Error bars represent standard error. [file MPP-26-e70170-s005.pdf]

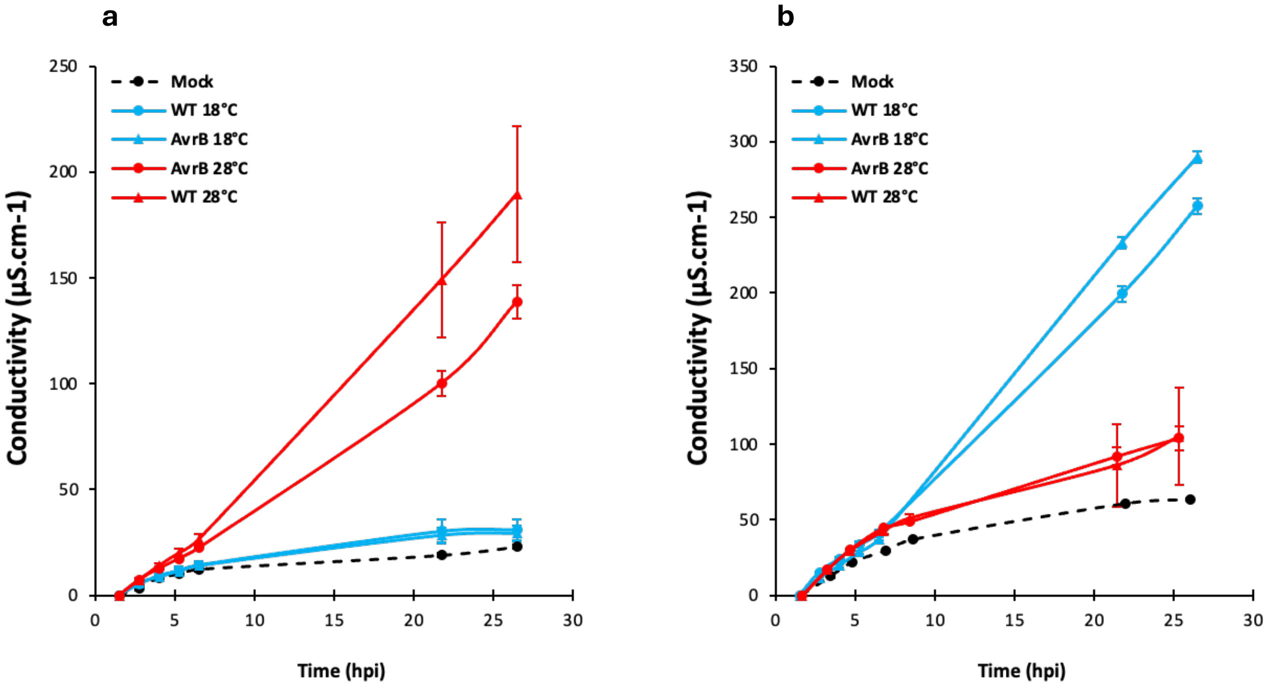

Figure S4. Electrolyte leakage curves over time obtained post-infiltration for CC0094 in *A. thaliana* Col-0 (a) and CRA-FRU 8.43 in *Actinidia arguta* (b). Infiltrated leaf disks were incubated at 18°C (blue) and 28°C (red). Conductivity following infiltration of the mock treatment (10mM $MgCl_2$) and incubation at 18°C is represented by the black dotted line. Data represent one single representative biological replicate with all strains and temperature conditions evaluated simultaneously with three technical replicates each. Error bars represent standard error.
