# Supplementary material for: Some Like it Hot: Efficiency of the Type III Secretion System has Multiple Thermosensitive Behaviours in the Pseudomonas syringae Complex
Source: Mol Plant Pathol. 2025 Dec 10;26(12):e70170. doi: 10.1111/mpp.70170 (PMC12696027; doi:10.1111/mpp.70170)
Supplement: Supplementary file 5 — Data S1: mpp70170‐sup‐0005‐SupplementaryMethods.pdf. [file MPP-26-e70170-s007.pdf]

Supplementary Material and Methods

Bacterial strain selection

Selected strains represented four phylogroups/clades (1a, 1b, 2d, and 3a). The geographical origin and isolation source of the strains varied, encompassing annual crops (*e.g.* M6, T1) (Debener et al., 1991; Whalen et al., 1991), woody plants (*e.g.* J35, CRA-FRU 8.43, MAFF302273) (Ferrante and Scortichini, 2010; Sawada et al., 1999; Takikawa et al., 1989), and environmental reservoirs like rivers or snow (*e.g.* LAB0041, USA007) (Berge et al., 2014; Morris et al., 2010). Genomic relatedness within a phylogroup was also considered, aiming for a balance of very divergent and closely related strains, such as the three 'CC' strains (CC0073, CC0094, and CC1498) that are quasi-clones (ANI > 99.83%) (Monteil et al., 2016; Morris et al., 2000; Morris et al., 2008). Model strains DC3000, B728a and 1448A were also included in the set as there is a plethora of information about their biology (Cuppels, 1986; Loper and Lindow, 1987; Taylor et al., 1996).

Ion-leakage experiments

Strains expressing *avrB* were streaked from glycerol stocks on King's B agar (KB) (King et al., 1954) – supplemented with kanamycin at 50ug/mL and rifampicin at 50µg/mL for DC3000 and B728a strains. After 48h of incubation at 28°C, single colonies of each strain were picked and grown overnight in liquid selective KB medium, with agitation at 190 rpm at 28°C. Similar procedure was followed for CC0094 WT (grown in non-selective KB). *A. thaliana* Col-0 plants were cultivated in a mixture of peat soil and perlite (4/1, v/v) in a growth chamber set at 24°C/21.5°C thermoperiod and 8 h light–16 h dark photoperiod with 70% of relative humidity for 6 to 8 weeks. Three healthy plants with six 5-mm leaf disks detached from a same plant with a cork borer were used for each experimental condition. To recognize the plant they were detached from, leaf disks were marked with a colored felt-tip and were then transferred into a 50mL tube containing 10mM $MgCl_2$ solution during the time

of bacterial suspension preparation. Ten mL of fresh bacterial suspensions were centrifuged (5min at 4500g at room temperature) and pellets were washed three times in 10mL of sterile 10mM $MgCl_2$ solution. Optical density at 600nm was measured by spectrophotometer (Evolution™, ThermoFisher Scientific, USA) and bacterial cells were finally resuspended in sterile 10mM $MgCl_2$ solution to reach 10^8 CFU/mL (OD_{600nm} = 0.1; final volume = 30mL). Leaf disks were infiltrated with the bacterial suspensions using vacuum (applied using a 50mL syringe or a vacuum chamber). Sterile 10mM $MgCl_2$ solution or *hrp*-inducing medium (HIM; using 27mM glycerol as carbon source; pH 5.5) (Huynh et al., 1989) were used as mock treatments. Infiltrated leaf disks were rinsed twice for 15 min in 40mL of milliQ water under gentle agitation (90 rpm) at room temperature to eliminate suspension/mock solution trace and ions leaked due to mechanical damage. Infiltrated leaf disks were then distributed into 12-well plate containing 2mL of milliQ water per well to have: (i) six disks per well, (ii) two disks from each of the three different plants per well, (iii) three identical wells (one plate column) per each experimental modality. Initial conductivity values ($\mu S.cm^{-1}$) in each well was measured prior plates incubation (120 rpm; constant light of 80 $\mu mol.m^{-2}.s^{-1}$) at different temperatures (18°C, 24°C and 28°C) using a compact conductometer (LAQUAtwin EC-11, HORIBA Scientific, Japan). Conductivity increase was measured every 1.5-2 hours during the first 8 hours of the experiment, and then twice between 18- and 24-hours post-inoculation (hpi), obtaining from 6 to 9 conductivity measures on average per well. Each experimental modality (*i.e.* one specific *avrB*-expressing strain infiltrated and then incubated at a certain temperature) was reproduced at least three times (at least 3 `biological replicates' or `replicated experiments').

To assess the hypersensitive response induction in kiwifruit, adult plants of *Actinidia arguta* var. Kens Red were recovered from a local nursery (Vivai Guardini, Pescantina, Verona, Italy) and maintained in pots in a greenhouse following natural environmental conditions. Strains CRA-FRU 8.43 WT and

avrB were streaked from glycerol stocks on LB agar and incubated at 22°C for 48h. Cells were recovered from this plate and spread on fresh LB agar plates and incubated overnight at 22°C. Cells were finally scraped from plates and suspended in a 10 mM $MgCl_2$ solution to reach 10^9 CFU/mL (OD_{600nm} = 1). Leaf disks (8 mm) were made from young leaves of a single *A. arguta* var. Kens Red plant and vacuum infiltrated using a syringe following the same protocol used for *A. thaliana*.

Effector repertoire characterization

The effector repertoires of ten *P. syringae* strains from our set were already publicly available (Dillon et al., 2019; Laflamme et al., 2020). We characterized the effector repertoires of the remaining strains (LAB0041, CC0073, CC1498) from their draft genomes (provided by Dr. Cindy Morris). The genomes were analyzed following a custom bioinformatic pipeline created with the help of the Nextflow framework (Tommaso et al., 2017). The pipeline was composed of several steps: i) the FastANI tool was used for rapid many-to-many ANI calculation (Jain et al., 2018); ii) BUSCO was used to assess both the genome quality and protein annotations quality (Manni et al., 2021); iii) the Prokka pipeline was used for the protein annotation (Seemann, 2014). The three genome protein annotations produced were aligned to the amino acid sequences of 529 T3SE proteins (obtained from the *Pseudomonas syringae* Type III Effector Compendium, PsyTEC) (Laflamme et al., 2020), list available at: https://guttman.csb.utoronto.ca/files/2023/07/PsyTEC_Public_Resource.xlsx) in a reciprocal best-hit (RBH) alignment via BLASTP, run with an e-value threshold of 1e-24 (Altschul et al., 1997; Daubin et al., 2002; Eisen, 2000; Hernández-Salmerón and Moreno-Hagelsieb, 2020). The alignment outputs were further refined with an R script, which kept, for each genome, only the unique proteins present in the T3SE list with a minimum RBH alignment coverage of 60% and a minimum identity score of 95%. These resulting proteins were considered as candidate effectors for the relative genome and were used to produce a presence/absence effector table for the 3 genomes,

which was then merged with the existing one for the 10 previously characterized strains. Information was synthetized to present family-level classified effector repertoires.

Statistical analysis (Linear Mixed-effects Models)

This allowed us to account for the important variability among the diverse experimental replicates of a same modality and, most importantly, to consider the entire time course of electrolyte leakage, rather than focusing solely on isolated time points. The mathematical representation of LMM is: $Y = X\beta + Z\upsilon + \varepsilon$; where Y represents the dependent variable (the measured feature, *i.e.* conductivity increase), $X\beta$ stands for the fixed effects (*i.e.* the potential explanatory variables we want to assess the effect on Y), $Z\upsilon$ stands for the random effects (all the unknown parameters that may be responsible for the huge variability among the different replicated experiments and which we are not directly interested to), and ε represents the residual. Using LMM in RStudio, we evaluated (i) the strain effect (*i.e.* if the ion-leakage following *avrB*-expressing strain infiltration was significantly different from the ion-leakage due to mock treatment infiltration) to categorize each strain as 'HR-inducer' or 'non-inducer' at each of the tested temperatures; and (ii) the temperature effect (*i.e.* if the ion-leakage following *avrB*-expressing strain infiltration was significantly different as the incubation temperature changed) on the strains considered as HR-inducers at least at two of the tested temperatures. In this way we estimated the values of model parameters and the associated p-values.

Supplementary References

Altschul, S.F., Madden, T.L., Schäffer, A.A., Zhang, J., Zhang, Z., Miller, W., et al. (1997) Gapped BLAST and PSI-BLAST: a new generation of protein database search programs. *Nucleic acids research*, 25, 3389–3402.

Berge, O., Monteil, C.L., Bartoli, C., Chandeysson, C., Guilbaud, C., Sands, D.C., et al. (2014) A user's guide to a data base of the diversity of *Pseudomonas syringae* and its application to classifying strains in this phylogenetic complex. *PLoS ONE*, 9, e105547.

Cuppels, D.A. (1986) Generation and characterization of Tn5 insertion mutations in *Pseudomonas syringae* pv. *tomato*. *Applied and Environmental Microbiology*, 51, 323–327.

Daubin, V., Gouy, M. & Perrière, G. (2002) A phylogenomic approach to bacterial phylogeny: evidence of a core of genes sharing a common history. *Genome research*, 12, 1080–1090.

Debener, T., Lehnackers, H., Arnold, M. & Dangl, J.L. (1991) Identification and molecular mapping of a single *Arabidopsis thaliana* locus determining resistance to a phytopathogenic *Pseudomonas syringae* isolate. *The Plant Journal*, 1, 289–302.

Dillon, M.M., Almeida, R.N.D., Laflamme, B., Martel, A., Weir, B.S., Desveaux, D., et al. (2019) Molecular evolution of *Pseudomonas syringae* type III secreted effector proteins. *Frontiers in plant science*, 10, 418.

Eisen, J.A. (2000) Assessing evolutionary relationships among microbes from whole-genome analysis. *Current opinion in microbiology*, 3, 475–480.

Ferrante, P. & Scortichini, M. (2010) Molecular and phenotypic features of *Pseudomonas syringae* pv. *actinidiae* isolated during recent epidemics of bacterial canker on yellow kiwifruit (*Actinidia chinensis*) in central Italy. *Plant pathology*, 59, 954–962.

Hernández-Salmerón, J.E. & Moreno-Hagelsieb, G. (2020) Progress in quickly finding orthologs as reciprocal best hits: comparing blast, last, diamond and MMseqs2. *BMC genomics*, 21, 741.

Huynh, T.V., Dahlbeck, D. & Staskawicz, B.J. (1989) Bacterial blight of soybean: regulation of a pathogen gene determining host cultivar specificity. *Science*, 245, 1374–1377.

Jain, C., Rodriguez-R, L.M., Phillippy, A.M., Konstantinidis, K.T. & Aluru, S. (2018) High throughput ANI analysis of 90K prokaryotic genomes reveals clear species boundaries. *Nature communications*, 9, 5114.

King, E.O., Ward, M.K. & Raney, D.E. (1954) Two simple media for the demonstration of pyocyanin and fluorescin. *The journal of laboratory and clinical medicine*, 44, 301–307.

Laflamme, B., Dillon, M.M., Martel, A., Almeida, R.N.D., Desveaux, D. & Guttman, D.S. (2020) The pan-genome effector-triggered immunity landscape of a host-pathogen interaction. *Science*, 367, 763–768.

Loper, J.E. & Lindow, S.E. (1987) Lack of evidence for *in situ* fluorescent pigment production by *Pseudomonas syringae* pv. *syringae* on bean leaf surfaces. *Phytopathology*, 77, 1449.

Manni, M., Berkeley, M.R., Seppey, M., Simão, F.A. & Zdobnov, E.M. (2021) BUSCO update: Novel and streamlined workflows along with broader and deeper phylogenetic coverage for scoring of eukaryotic, prokaryotic, and viral genomes. *Molecular biology and evolution*, 38, 4647–4654.

Monteil, C.L., Yahara, K., Studholme, D.J., Mageiros, L., Méric, G., Swingle, B., et al. (2016) Population-genomic insights into emergence, crop adaptation and dissemination of *Pseudomonas syringae* pathogens. *Microbial genomics*, 2, e000089.

Morris, C.E., Glaux, C., Latour, X., Gardan, L., Samson, R. & Pitrat, M. (2000) The relationship of host range, physiology, and genotype to virulence on cantaloupe in *Pseudomonas syringae* from cantaloupe blight epidemics in France. *Phytopathology*, 90, 636–646.

Morris, C.E., Sands, D.C., Vanneste, J.L., Montarry, J., Oakley, B., Guilbaud, C., et al. (2010) Inferring the evolutionary history of the plant pathogen *Pseudomonas syringae* from its biogeography in headwaters of rivers in North America, Europe, and New Zealand. *mBio*, 1. https://doi.org/10.1128/mBio.00107-10.

Morris, C.E., Sands, D.C., Vinatzer, B.A., Glaux, C., Guilbaud, C., Buffière, A., et al. (2008) The life history of the plant pathogen *Pseudomonas syringae* is linked to the water cycle. *The ISME Journal*, 2, 321–334.

Sawada, H., Suzuki, F., Matsuda, I. & Saitou, N. (1999) Phylogenetic analysis of *Pseudomonas syringae* pathovars suggests the horizontal gene transfer of *argK* and the evolutionary stability of *hrp* gene cluster. *Journal of Molecular Evolution*, 49, 627–644.

Seemann, T. (2014) Prokka: rapid prokaryotic genome annotation. *Bioinformatics*, 30, 2068–2069.

Takikawa, Y., Serizawa, S., Ichikawa, T., Tsuyumu, S. & Goto, M. (1989) *Pseudomonas syringae* pv. *actinidiae* pv. nov.: the causal bacterium of canker of kiwifruit in Japan. *Japanese Journal of Phytopathology*, 55, 437–444.

Taylor, J.D., Teverson, D.M., Allen, D.J. & Pastor-Corrales, M.A. (1996) Identification and origin of races of *Pseudomonas syringae* pv. *phaseolicola* from Africa and other bean growing areas. *Plant Pathology*, 45, 469–478.

Tommaso, D., Chatzou, P., Floden, M., Barja, E.W., Palumbo, P.P. & Notredame, E. (2017) Nextflow enables reproducible computational workflows. *Nature Biotechnology*, 35, 316–319.

Whalen, M.C., Innes, R.W., Bent, A.F. & Staskawicz, B.J. (1991) Identification of *Pseudomonas syringae* pathogens of *Arabidopsis* and a bacterial locus determining avirulence on both *Arabidopsis* and soybean. *The Plant Cell*, 3, 49.
