# Supplementary material for: Some Like it Hot: Efficiency of the Type III Secretion System has Multiple Thermosensitive Behaviours in the Pseudomonas syringae Complex
Source: Mol Plant Pathol. 2025 Dec 10;26(12):e70170. doi: 10.1111/mpp.70170 (PMC12696027; doi:10.1111/mpp.70170)
Supplement: Supplementary file 6 — Table S1: Set of selected P. syringae strains. [file MPP-26-e70170-s008.pdf]

Table S1. Set of selected *P. syringae* strains.

PG	STRAIN	FULL NAME	ALIAS IN COLLECTIONS	ISOLATION SOURCE	COUNTRY	YEAR	REFERENCE
1a	DC3000	*P. syringae* pv. *tomato*	CFBP 7438, ICMP 18429	*Solanum lycopersicum*	United-Kingdom	1960	(Cuppels, 1986)
	M6	*P. syringae* pv. *maculicola*	CFBP 4314, NCPPB 1766, LMG 5560	*Brassica oleracea* var. Botrytis	United-Kingdom	1965	(Debener et al., 1991)
	T1	*P. syringae* pv. *tomato*	-	*Solanum lycopersicum*	Canada	1986	(Whalen et al., 1991)
	LAB0041	*P. syringae*	-	Epilithic biofilm	France	2009	(Berge et al., 2014)
1b	J35	*P. syringae* pv. *actinidiae*	CFBP 4909, ICMP 9617, NCPPB 3739	*Actinidia deliciosa* cv. Hayward	Japan	1984	(Takikawa et al., 1989)
	CRA-FRU 8.43	*P. syringae* pv. *actinidiae*	-	*Actinidia chinensis* cv. Hort16	Italy	2008	(Ferrante and Scortichini, 2010)
	USA007	*P. syringae*	CFBP 8574	Creek water	United States of America	2007	(Morris et al., 2010)
2d	CC0073	*P. syringae* pv. *aptata*	CFBP 5430	*Cucumis melo*	France	1997	(Morris et al., 2000)
	CC0094	*P. syringae* pv. *aptata*	CFBP 8529	*Cucumis melo*	France	1997	(Morris et al., 2000)
	CC1498	*P. syringae*	-	Snowfall	France	2006	(Morris et al., 2008)
	B728a	*P. syringae* pv. *syringae*	CFBP 8502, ICMP 18427, NCPPB 4487, LMG 26717	*Phaseolus vulgaris*	United States of America	1987	(Loper and Lindow, 1987)
	MAFF302273	*P. syringae* pv. *aceris*	CFBP 2339, ICMP 2802, NCPPB 958, LMG 2106	*Acer* sp.	United States of America	1939	(Sawada et al., 1999)
3a	1448A	*P. syringae* pv. *phaseolicola*	CFBP 7087, NCPPB 4478	*Phaseolus vulgaris*	Ethiopia	1985	(Taylor et al., 1996)

Berge, O., Monteil, C.L., Bartoli, C., Chandeysson, C., Guilbaud, C., Sands, D.C., et al. (2014) A user's guide to a data base of the diversity of *Pseudomonas syringae* and its application to classifying strains in this phylogenetic complex. *PLoS ONE*, 9, e105547.

Cuppels, D.A. (1986) Generation and characterization of Tn5 insertion mutations in *Pseudomonas syringae* pv. *tomato*. *Applied and Environmental Microbiology*, 51, 323–327.

Debener, T., Lehnackers, H., Arnold, M. & Dangl, J.L. (1991) Identification and molecular mapping of a single *Arabidopsis thaliana* locus determining resistance to a phytopathogenic *Pseudomonas syringae* isolate. *The Plant Journal*, 1, 289–302.

Ferrante, P. & Scortichini, M. (2010) Molecular and phenotypic features of *Pseudomonas syringae* pv. *actinidiae* isolated during recent epidemics of bacterial canker on yellow kiwifruit (*Actinidia chinensis*) in central Italy. *Plant pathology*, 59, 954–962.

Loper, J.E. & Lindow, S.E. (1987) Lack of evidence for *in situ* fluorescent pigment production by *Pseudomonas syringae* pv. *syringae* on bean leaf surfaces. *Phytopathology*, 77, 1449.

Morris, C.E., Glaux, C., Latour, X., Gardan, L., Samson, R. & Pitrat, M. (2000) The relationship of host range, physiology, and genotype to virulence on cantaloupe in *Pseudomonas syringae* from cantaloupe blight epidemics in France. *Phytopathology*, 90, 636–646.

Morris, C.E., Sands, D.C., Vanneste, J.L., Montarry, J., Oakley, B., Guilbaud, C., et al. (2010) Inferring the evolutionary history of the plant pathogen *Pseudomonas syringae* from its biogeography in headwaters of rivers in North America, Europe, and New Zealand. *mBio*, 1. https://doi.org/10.1128/mBio.00107-10.

Morris, C.E., Sands, D.C., Vinatzer, B.A., Glaux, C., Guilbaud, C., Buffière, A., et al. (2008) The life history of the plant pathogen *Pseudomonas syringae* is linked to the water cycle. *The ISME Journal*, 2, 321–334.

Sawada, H., Suzuki, F., Matsuda, I. & Saitou, N. (1999) Phylogenetic analysis of *Pseudomonas syringae* pathovars suggests the horizontal gene transfer of *argK* and the evolutionary stability of *hrp* gene cluster. *Journal of Molecular Evolution*, 49, 627–644.

Takikawa, Y., Serizawa, S., Ichikawa, T., Tsuyumu, S. & Goto, M. (1989) *Pseudomonas syringae* pv. *actinidiae* pv. nov.: the causal bacterium of canker of kiwifruit in Japan. *Japanese Journal of Phytopathology*, 55, 437–444.

Taylor, J.D., Teverson, D.M., Allen, D.J. & Pastor-Corrales, M.A. (1996) Identification and origin of races of *Pseudomonas syringae* pv. *phaseolicola* from Africa and other bean growing areas. *Plant Pathology*, 45, 469–478.

Whalen, M.C., Innes, R.W., Bent, A.F. & Staskawicz, B.J. (1991) Identification of *Pseudomonas syringae* pathogens of *Arabidopsis* and a bacterial locus determining avirulence on both *Arabidopsis* and soybean. *The Plant Cell*, 3, 49.
