# Supplementary material for: Some Like it Hot: Efficiency of the Type III Secretion System has Multiple Thermosensitive Behaviours in the Pseudomonas syringae Complex
Source: Mol Plant Pathol. 2025 Dec 10;26(12):e70170. doi: 10.1111/mpp.70170 (PMC12696027; doi:10.1111/mpp.70170)
Supplement: Supplementary file 7 — Table S2: Matrix of effectors found among the P. syringae strains. Effector repertoires of all strains used in this study were already available publicly, with the exception of CC0073, CC1498 and LAB0041 whose repertoires were characterised following the same procedure, as described (Laflamme et al. 2020). [file MPP-26-e70170-s003.pdf]

Table S2. Matrix of effectors found among the *P. syringae* strains. Effector repertoires of all strains used in this study were already available publicly, with the exception of CC0073, CC1498 and LAB0041 whose repertoires were characterized following the same procedure, as described (Laflamme et al., 2020[b]).

Effector family[a]	DC3000	M6	T1	LAB0041	J35	CRA-FRU 8.43	USA007	CC0073	CC0094	CC1498	B728a	MAFF302273	1448A
AvrA	0	0	1	0	0	0	0	0	0	0	0	0	0
AvrB	0	0	0	0	1	1	0	0	0	0	1	0	3
AvrE	1	1	1	1	1	1	1	2	1	2	1	1	1
AvrPto	1	0	0	0	1	1	0	0	0	0	0	0	0
AvrRpm	0	0	0	0	2	1	0	0	0	0	1	1	0
AvrRpt	0	0	1	0	0	0	0	0	0	0	0	0	0
HopA	1	1	2	1	0	2	1	0	0	0	0	0	0
HopB	3	2	2	1	1	2	1	2	2	1	1	2	2
HopC	1	0	2	1	0	0	0	0	0	0	0	0	0
HopD	3	1	1	0	2	2	0	0	0	0	0	0	1
HopE	1	0	0	0	0	0	0	0	0	0	0	0	0
HopF	1	1	1	2	3	3	1	0	0	0	0	1	1
HopG	1	0	0	0	0	0	0	0	0	0	0	0	1
HopH	1	0	1	0	0	1	0	1	1	1	1	1	0
HopI	1	1	1	0	1	1	1	1	1	1	1	1	1
HopK	1	1	0	0	0	0	0	0	0	0	0	0	1
HopL	1	1	1	1	0	0	1	0	0	0	1	0	0
HopM	1	1	2	1	1	1	1	1	1	1	1	1	1
HopN1	1	1	0	0	1	1	0	0	0	0	0	0	0
HopO	5	3	5	1	0	0	2	0	0	0	0	0	0
HopQ	1	1	1	0	1	1	0	0	0	0	0	0	1
HopR	1	1	1	1	1	1	1	0	0	0	0	0	1
HopS	1	1	1	2	1	1	1	0	0	0	0	0	0
HopT	2	1	2	0	0	0	1	0	0	0	0	0	0
HopU	1	1	0	0	0	0	0	0	0	0	0	0	0
HopV	1	0	0	2	0	1	0	0	0	0	0	0	1
HopW	0	0	2	1	3	2	0	1	1	1	1	1	3
HopX	1	1	0	0	1	0	1	0	0	0	1	0	1
HopY	1	1	1	3	1	1	1	0	0	0	0	0	0
HopZ	0	0	0	0	0	1	0	0	0	0	0	1	0
HopAA	2	1	1	1	1	3	1	4	1	4	1	1	2
HopAB	1	1	1	0	1	0	1	2	1	2	1	0	3
HopAD	1	0	0	0	0	0	0	0	0	0	0	0	0
HopAF	1	1	1	3	1	1	1	0	0	0	1	1	1
HopAG	2	1	1	2	1	2	1	1	1	1	1	2	0
HopAH	3	3	3	0	3	3	3	1	2	1	2	2	1
HopAI	1	1	1	2	2	1	1	2	1	2	1	2	0
HopAL	0	0	0	0	0	0	0	1	1	1	0	1	0
HopAM	2	0	0	0	1	1	0	0	0	0	0	0	0
HopAQ	1	0	0	0	0	0	0	0	0	0	0	0	0
HopAR	0	0	0	0	1	0	0	0	1	1	0	0	0
HopAS	2	2	1	2	1	1	1	0	0	0	0	0	1
HopAT	2	0	1	0	1	2	0	0	0	0	0	0	3
HopAU	0	0	0	0	1	1	0	0	0	0	0	0	1
HopAW	0	0	0	0	0	1	0	0	0	0	0	0	1
HopAZ	0	0	0	1	1	1	0	0	0	0	0	0	0
HopBC	0	0	0	0	0	0	0	0	0	0	0	1	0
HopBE	0	0	0	0	0	0	0	0	0	0	0	1	0
HopBK	0	0	0	0	0	0	0	0	0	1	0	0	0
HopBM	2	0	0	0	0	0	0	0	0	0	0	0	0
HopBN	0	0	1	2	1	1	0	0	0	0	0	0	0
HopBO	0	0	0	0	1	0	1	0	0	0	0	0	0
HopBP	0	0	0	0	1	1	2	0	0	0	1	0	0
HopBQ	0	0	0	0	2	0	0	0	0	0	0	0	0

[a] For each effector family identified in a given strain, we indicated the number of clusters present in the genome (from 1 to 5). The intensity of the colour scale follows increasing cluster numbers.

[b] Laflamme, B., Dillon, M.M., Martel, A., Almeida, R.N.D., Desveaux, D. & Guttman, D.S. (2020) The pan-genome effector-triggered immunity landscape of a host-pathogen interaction. *Science*, 367, 763–768.
