# Supplementary material for: Some Like it Hot: Efficiency of the Type III Secretion System has Multiple Thermosensitive Behaviours in the Pseudomonas syringae Complex
Source: Mol Plant Pathol. 2025 Dec 10;26(12):e70170. doi: 10.1111/mpp.70170 (PMC12696027; doi:10.1111/mpp.70170)
Supplement: Supplementary file 8 — Table S3: Known interaction outcomes of P. syringae WT and avrB‐expressing strains in Arabidopsis thaliana Col‐0. [file MPP-26-e70170-s004.pdf]

Table S3. Known interaction outcomes of *P. syringae* WT and *avrB*-expressing strains in *Arabidopsis thaliana* Col-0.

Phylogroup	Species	Strain	Interaction outcome WT strain / *A. thaliana* Col-0	Interaction outcome *avrB*-expressing strain / *A. thaliana* Col-0
1a	*P. syringae* pv. *tomato*	DC3000	Compatible/disease (no HR) (Preston, 2000; Xin and He, 2013)	Avirulent/host resistance (HR) (Innes et al., 1993; Mackey et al., 2002; Mudgett, 2005)
	P. syringae pv. *maculicola*	M6	Compatible/disease (no HR) (Debener et al., 1991)	Avirulent/host resistance (electrolyte leakage indicative of an HR) (This study)
	P. syringae pv. *tomato*	T1	Non-host resistance type II (HR) (Ishiga et al., 2011)	Non-host resistance type II (electrolyte leakage indicative of an HR) (This study)
	P. syringae pv. *actinidiae*	CRA-FRU 8.43	Non-host resistance type I (no HR) (Jayaraman et al., 2017)	Non-host resistance type II (HR) (Puttilli et al., 2022)
2d	*P. syringae* pv. *aptata*	CC0094	Non-host resistance type II (HR) (electrolyte leakage indicative of an HR) (This study)	Non-host resistance type II (electrolyte leakage indicative of an HR) (This study)
	P. syringae pv. *syringae*	B728a	Non-host resistance type I (no HR) (Vinatzer et al., 2006)	Non-host resistance type II (electrolyte leakage indicative of an HR) (This study)
3a	*P. syringae* pv. *phaseolicola*	1448A	Non-host resistance type I (no HR) (Ham et al., 2007)	Non-host resistance type II (electrolyte leakage indicative of an HR) (This study)

Debener, T., Lehnackers, H., Arnold, M. & Dangl, J.L. (1991) Identification and molecular mapping of a single *Arabidopsis thaliana* locus determining resistance to a phytopathogenic *Pseudomonas syringae* isolate. *The Plant Journal*, 1, 289–302.

Ham, J.H., Kim, M.G., Lee, S.Y. & Mackey, D. (2007) Layered basal defenses underlie non-host resistance of *Arabidopsis* to *Pseudomonas syringae* pv. *phaseolicola*. *The Plant journal: for cell and molecular biology*, 51, 604–616.

Innes, R.W., Bisgrove, S.R., Smith, N.M., Bent, A.F., Staskawicz, B.J. & Liu, Y.C. (1993) Identification of a disease resistance locus in *Arabidopsis* that is functionally homologous to the RPG1 locus of soybean. *The Plant journal: for cell and molecular biology*, 4, 813–820.

Ishiga, Y., Ishiga, T., Uppalapati, S.R. & Mysore, K.S. (2011) *Arabidopsis* seedling flood-inoculation technique: a rapid and reliable assay for studying plant-bacterial interactions. *Plant methods*, 7, 32.

Jayaraman, J., Choi, S., Prokchorchik, M., Choi, D.S., Spiandore, A., Rikkerink, E.H., et al. (2017) A bacterial acetyltransferase triggers immunity in *Arabidopsis thaliana* independent of hypersensitive response. *Scientific reports*, 7, 3557.

Mackey, D., Holt, B.F., 3rd, Wiig, A. & Dangl, J.L. (2002) RIN4 interacts with *Pseudomonas syringae* type III effector molecules and is required for RPM1-mediated resistance in *Arabidopsis*. *Cell*, 108, 743–754.

Mudgett, M.B. (2005) New insights to the function of phytopathogenic bacterial type III effectors in plants. *Annual review of plant biology*, 56, 509–531.

Preston, G.M. (2000) *Pseudomonas syringae* pv. *tomato* : right pathogen, right plant, right time. *Molecular Plant Pathology*, 1, 263–275.

Puttilli, M.R., Danzi, D., Correia, C., Brandi, J., Cecconi, D., Manfredi, M., et al. (2022) Plant signals anticipate the induction of the type III secretion system in *Pseudomonas syringae* pv. *actinidiae*, facilitating efficient temperature-dependent effector translocation. *Microbiology spectrum*, 10, e0207322.

Vinatzer, B.A., Teitzel, G.M., Lee, M.-W., Jelenska, J., Hotton, S., Fairfax, K., et al. (2006) The type III effector repertoire of *Pseudomonas syringae* pv. *syringae* B728a and its role in survival and disease on host and non-host plants. *Molecular microbiology*, 62, 26–44.

Xin, X.F. & He, S.Y. (2013) *Pseudomonas syringae* pv. *tomato* DC3000: a model pathogen for probing disease susceptibility and hormone signaling in plants. *Annual review of phytopathology*, 51, 473–498.
